# Myelin oligodendrocyte glycoprotein (MOG) antibody-associated encephalitis induced by *Mycoplasma pneumoniae* infections

**DOI:** 10.1186/s13052-024-01768-w

**Published:** 2024-09-27

**Authors:** Yan-Ru Liu, Xiang-Dong Zeng, Ying Xiong

**Affiliations:** 1 Department of Pediatric Chengdu Women’s and Children’s Central Hospital, School of Medicine, University of Electronic Science and Technology of China, Chengdu, China; 2 Department of Pediatric Pulmonology and Immunology, West China Women’s and Children’s Hospital: Sichuan University West China Second University Hospital, Sichuan Province, Chengdu, China

**Keywords:** MOG-IgG antibodies, *Mycoplasma* *pneumoniae* infections, Children

## Abstract

**Background:**

This study aims to report the phenomenon of Myelin oligodendrocyte glycoprotein antibody-associated encephalitis induced by Mycoplasma *pneumoniae* infections and promote the potential benefits of combining early immunotherapy and anti*-M—pneumoniae* therapy for these patients.

**Methods:**

Three children with MOG-IgG-associated encephalitis due to M. *pneumoniae* infections who were treated at our hospital from September to November 2023 were included in the study. We investigated and analyzed the background and clinical features of these patients.

**Results:**

Three patients developed headaches, seizures, and/or other neurological manifestations, elevated mononuclear cells in cerebrospinal fluid, intracranial lesions on cranial magnetic resonance imaging (MRI), and positive MOG-IgG in serum, within 10–14 days. They were diagnosed with MOG-IgG-associated encephalitis due to M. *pneumoniae* infections, the treatment consisted of intravenous immunoglobulin, glucocorticoid, and erythromycin, then they were completely recovered.

**Conclusion:**

Mycoplasma pneumoniae (*M. pneumoniae*) infections can cause oligodendrocyte glycoprotein (MOG) antibody-associated encephalitis. The recognition of this condition will promote the potential benefits of combining early immunotherapy and anti*-M. pneumoniae* therapy for patients with MOG-IgG-associated encephalitis.

## Introduction

Mycoplasma pneumoniae (*M. pneumoniae*) is one of the most common causes of community-acquired pneumonia [1]. In addition to affecting the respiratory tract, it also affects the cardiovascular, digestive, dermatological, neurological, and hematological systems [2]. However, Myelin oligodendrocyte glycoprotein Immunoglobulin G (MOG-IgG) -associated encephalitis due to *M. pneumoniae* infections was rarely reported previously. Here we describe the clinical course and management of the cases with MOG-IgG associated encephalitis induced by *M. pneumoniae* infections and look forward to expanding the spectrum of extrapulmonary manifestations of *M. pneumoniae* infections for early identification and potential benefits of early immunotherapy combined with anti-*M. pneumoniae* therapy in such patients.

## Methods

From September to November 2023, three patients with *M. pneumoniae* infections were admitted to our hospital to receive anti-*M. pneumoniae* treatment, during which they developed neurological symptoms such as headache, seizure, and confusion.

We investigated the background and clinical features of these patients including the age of infection onset, the respiratory symptoms, the pulmonary imaging, nasopharyngeal *M. pneumoniae* polymerase chain reaction (PCR), serum *M. pneumoniae* IgM, the period from infections to neurological symptoms, the neurological manifestations, the changes of cerebrospinal fluid(CSF), cranial magnetic resonance imaging(MRI), Electroencephalography (EEG), CSF and serological pathogens, Theo Serum MOG-IgG, the treatment and prognosis.

## Results

The clinical features of the three patients are summarized in Table 1. All patients presented with cough and fever in the early stage and neurological manifestations 10–14 days after the onset of cough and fever. Physical examination of all patients was normal, except for patient 3. with a positive Babinski sign. In all patients, complete blood cell count tests and c-reactive protein (CRP) were generally normal, EBV-IgM, adenovirus-IgM, respiratory syncytial virus-IgM, influenza virus-IgM and parainfluenza virus-IgM antibodies in serum were negative, blood autoantibodies were negative, abdomen CT were normal, glucose and protein levels in CSF were normal, pathogens antibodies (Herpes simplex virus(HSV), cytomegalovirus, Epstein-Barr virus(EBV), Japanese encephalitis virus and *M. pneumoniae,* etc.) and next-generation sequencing for pathogens (various common bacteria, fungi, viruses and *M. pneumoniae*, etc.) in CSF were negative, anti-NMDA receptor antibodies and oligoclonal bands in CSF were negative, AQP4 antibodies in serum were negative, Electroencephalography were normal except for the patient 1. had background rhythm slow(5-6 Hz), spinal cord MRI were normal.

**Table 1:** The clinical features of the three patients

Patient	Patient 1	Patient 2	Patient 3
Age(yrs), Sex	9,M	5,F	6,F
Symptoms upon admission (Prodromal symptom) and duration	Fever and cough, 2d	Fever and cough, 3d	Fever and cough, 4d
Neurological manifestations and days after admission	Headache, Vomiting, Orbital pain, 8d	Headache, Vomiting, T Seizure, 11d	Seizure, hypersomnia, positive Babinski sign,8d
Nasopharyngeal MP PCR	Positive	Positive	Positive
Serum MP-IgM(reference < 1:80)	1:160	1:1280	1:640
Pulmonary X-ray	bronchitis	bronchitis	bronchitis
CSF			
LEU(reference < 15*10^6/L)/(mon%)	71 /(82%)	111/(90%)	55/(80%)
Protein(reference 150-450 mg/L)	404	372	293
Glucose(reference 2.5–4.5 mmol/L)	3.6	4.3	3.9
MP PCR	negative	negative	negative
Cranial MRI	Abnormal (Fig. 1 a)	Abnormal (Fig. 1b)	Abnormal (Fig. 1 c)
EEG	slow background	normal	normal
Serum MOG-IgG	1:320	1:32	1:320
Antibiotics, dose and duration	Erythromycin, 20 mg/kg.d, 10d	Erythromycin, 20 mg/kg.d, 14d	Erythromycin, 20 mg/kg.d, 10d
IVIG dose	1 g/kg.d × 2d	1 g/kg.d × 2d	1 g/kg.d × 2d
IV methylprednisolone dose and duration	20 mg/kg.d × 5 d	20 mg/kg.d × 5 d	20 mg/kg.d × 5 d
Follow-up			
Duration of Steroid	3 m	3 m	4 m
Clinical manifestation	completely recovered	completely recovered	completely recovered
EEG	normal	normal	normal
Cranial MRI (after discharge)	Normal (8 m)	Normal (6 m)	Normal (7 m)
Serum MOG-IgG (after discharge)	1:32 (4 m)/ 1:10(6 m) Negative(8 m)	1:10 (3 m) Negative(6 m)	1:10 (5 m) Negative(7 m)

*yrs* Yeas old, *M* Male, *F* Female, *MP* Mycoplasma pneumoniae, *d* Days, *LEU* Leukocyte, *mon* Mononuclear cells, *EEG* Electroencephalogram, *MRI* Magnetic resonance imaging, *m* Months, *IVIG* Intravenous immunoglobulin, *IV* Intravenous

**Fig. 1:**
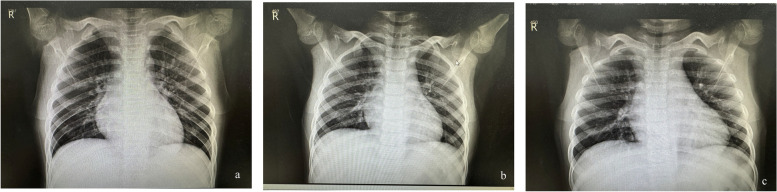
**a** patient 1. X-ray imaging of bilateral lung texture was disordered, enlarged, and blurred; **b** patient 2. X-ray imaging bilateral lung texture was disordered and enlarged; **c** patient 3. X-ray imaging bilateral lung texture was enlarged and blurred

**Fig. 2:**
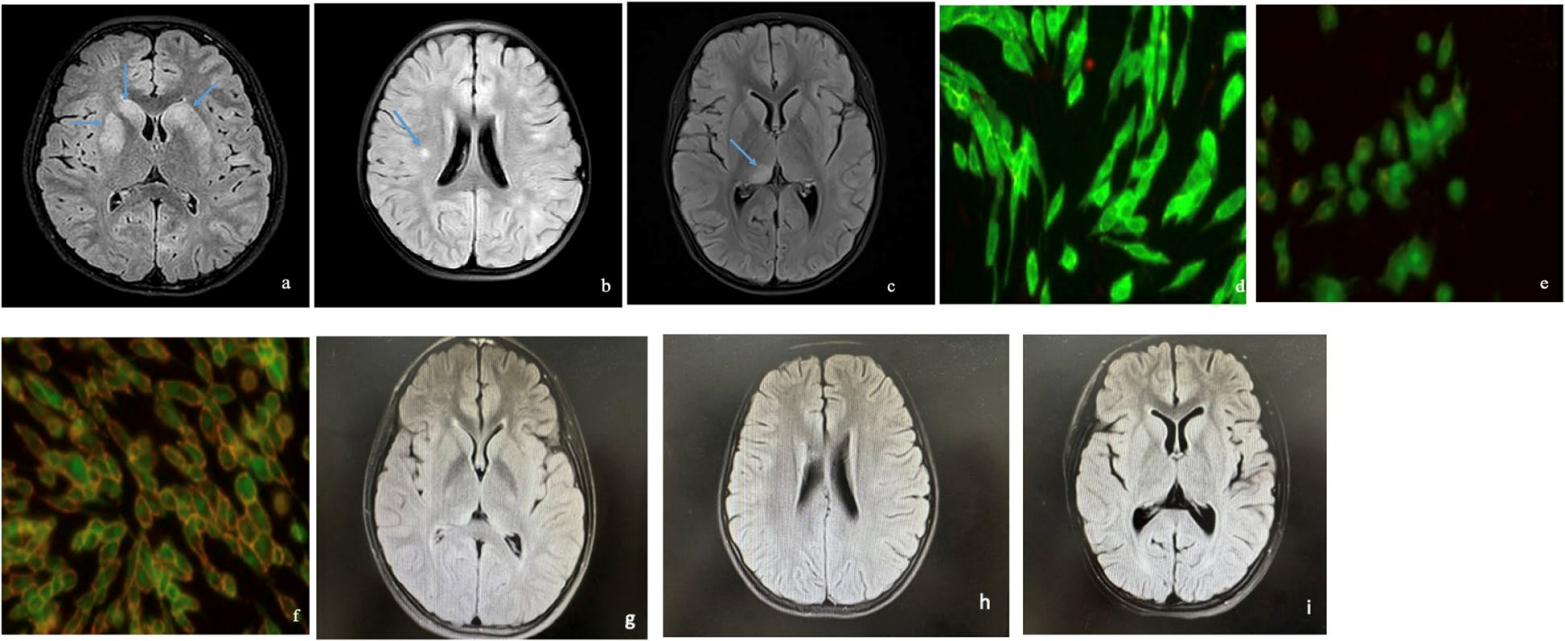
**a** patient 1. Axial FLAIR demonstrating hyperintensities on the bilateral caudate nucleus and lenticular nucleus; **b** patient 2. Axial FLAIR demonstrating hyperintensity on right centrum semiovale; **c** patient 3. Axial FLAIR demonstrating hyperintensity on right dorsal thalamus. **d** patient 1. MOG-IgG (green) in serum was detected by cell transfection assay; **e** patient 2. MOG-IgG (green) in serum was detected by cell transfection assay; **f** patient 3. MOG-IgG (green) in serum was detected by cell transfection assay; **g** patient 1. cranial MRI scan was normal 8 months after discharge; **h** patient 2. cranial MRI scan was normal 6 months after discharge; **i** patient 3. cranial MRI scan was normal 7 months after discharge. Ig: Immunoglobulin; MOG: Myelin oligodendrocyte glycoprotein

In all patients, serum *M. pneumoniae*-IgM antibodies were positive, nasopharyngeal *M. pneumoniae* PCR was positive, lung texture growing disorder on pulmonary X-ray imaging (Fig. 1 a-c), mononuclear cells were elevated in CSF, cranial MRI scan revealed intracranial lesions (Fig. 2 a-c), and MOG-IgG were positive in serum (Fig. 2 d-f).

They were diagnosed with MOG antibody-associated encephalitis due to *M. pneumoniae* infections, and the treatment consisted of intravenous immunoglobulin (IVIG, 2 g/kg over 2 d), glucocorticoid (intravenous injection of methylprednisolone at 20 mg/kg × 5 d, followed by oral prednisone at 2 mg/kg/d with tapering decrease in dose), and erythromycin (10 mg/kg once every 12h × 7-14d). During hospitalization, 5–7 days of glucocorticoid application, their clinical symptoms, and signs showed substantial recovery, the repeated CSF nucleated cells disappeared, nasopharyngeal *M. pneumoniae* PCR was negative, and then they were discharged home.

Until now, they were followed up for 6–8 months, all patients showed no signs or symptoms of neurological abnormalities, the serum MOG-IgG antibody titer decreased, cranial MRI scans were normal (Fig. 2 g-i), and prednisone has been discontinued.

## Discussion

*M. pneumoniae* most commonly causes a mild, self-limiting respiratory illness, including pharyngitis and acute bronchitis, pneumonia is less common [1]. Our cases were considered acute bronchitis because they had cough and fever, but no crackles were heard in the lungs, and the lung texture was coarse and disordered rather than lobar consolidation on chest X-ray. In addition to affecting the respiratory system,

*M. pneumoniae* may also cause a wide variety of extrapulmonary diseases including almost all organs of the human body [2, 3], gastrointestinal involvement was the most common extrapulmonary manifestation, represented mainly by vomiting, diarrhea, and abdominal pain, the second most frequent extrapulmonary manifestations were the dermatological ones, consisting mostly of skin rash [4]. Cardiovascular and neurological manifestations have also been described [5–7]. According to its anatomical classification, the manifestations of neurological can be broadly classified into brain disorders represented by encephalitis [8], spinal cord disorders represented by transverse myelitis [9], and peripheral nerve disorders represented by Guillain-Barré syndrome [10].

Encephalitis is the most frequent central nervous system manifestation of *M. pneumoniae* infections in children [11, 12], but MOG-IgG-associated encephalitis induced by *M. pneumoniae* infections has been rarely reported previously. MOG-IgG-associated disorders comprise a wide spectrum of syndromes ranging from acute-disseminated encephalomyelitis predominantly (ADEM) in children to optic neuritis or myelitis mostly in adults [13, 14]. The current literature reports MOG-associated meningoencephalitis and acute disseminated encephalomyelitis (ADEM) after *M. pneumoniae* infections [15, 16], but MOG-IgG-associated encephalitis induced by *M. pneumoniae* infections has not been reported. Of note, because of the ubiquity of *M. pneumoniae* infections, inflammation of the CNS induced by *M. pneumoniae* infections should be vigilant when a patient with neurological symptoms and clues of *M. pneumoniae* infections. Because in such conditions, immunotherapy is needed in addition to antibiotic treatment.

In our case, the clinical manifestations, nasopharyngeal *M. pneumoniae* PCR, and serum *M. pneumoniae*-IgM supported the diagnosis of *M. pneumoniae* infections. Combined with the CSF mononuclear cells, MRI results (although the MRI of Patient 2 exhibited certain atypical features that deviate from the classical presentation of encephalitis, considering her clinical manifestation and the increase of monocyte in CSF, we still considered that she was encephalitis with white matter involvement), and serum MOG-IgG antibodies, we considered that the *M. pneumoniae* infections induced MOG-IgG-associated encephalitis.

We were considering that anti-MOG antibodies have been associated with relapsing ADEM in literature, which we have been focusing on during follow-up visits. We followed them up longitudinally for 6–8 months, and none of them presented with relapsing ADEM (no signs or symptoms of neurological abnormalities, the serum MOG-IgG antibodies were negative, cranial MRI scan were normal), and we will continue to focus on this issue during the follow-up visits.

The exact pathophysiological mechanisms are not completely known, concerning this, the literature has claimed that neurologic manifestations due to *M. pneumoniae* infection can be classified into three categories [7], the first is a direct type in which locally induced cytokines must play a role, the second is an indirect type in which immune modulation such as autoimmunity must play a role, and the third is a vascular occlusion type in which vasculitis and/or thrombosis with or without systemic hypercoagulable state must play a role.

Our cases may point towards the second indirect mechanism because they did not develop neurological symptoms until 7 days after *M. pneumoniae* infection and CSF next-generation sequencing for *M. pneumoniae* was negative [17]. The possible hypothesis of the indirect mechanism is that *M. pneumoniae* cytoplasm contains potent immunogenic substances, such as glycolipids and glycoproteins, which can stimulate the production of steroid-responsive autoantibodies directed against myelin proteins via molecular mimicry (confirmed based on the detection of pathogenic antibodies in serum and/or CSF) [12]. Of note, these mechanisms are not mutually exclusive, and in such patients, *M. pneumoniae* can trigger autoimmunity while also inducing direct damage to the local blood–brain barrier.

There are some limitations to our study. First, this is a single-center study with a small sample size, thus our results may not be fully representative of extrapulmonary neurological manifestations in children with *M. pneumoniae* infection. Moreover, as a retrospective study, due to the lack of some initial data, such as CSF and serum cytokine levels (IL6, Interleukin-8, IL18, Tumor necrosis factors, etc.), we were unable to accurately delineate its pathological mechanism. Future research should summarize more cases through clinical observation to clarify the possible association between MOG-IgG-associated neurological disorders and *M. pneumoniae*. Such studies will have a very positive clinical impact, particularly in guiding clinicians towards considering *M. pneumoniae* as a possible etiological factor in cases of unexplained encephalitis with positive MOG antibodies, which could advance the potential benefits of combining early immunotherapy and anti*-M. pneumoniae* therapy in these patients.

## Conclusion

Though not a common presentation, MOG-IgG-associated disorders induced by *M. pneumoniae* infections should be vigilant when a patient with neurological symptoms and clues of *M. pneumoniae* infections because of the ubiquity of *M. pneumoniae* infections, and in such conditions, immunotherapy is needed in addition to antibiotic treatment, which will promote the potential benefit of immunotherapy combined with anti*-M. pneumoniae* therapy to patients with MOG-IgG-associated disorders.

## Data Availability

The data supporting this study’s findings are available on request from the corresponding author. The data are not publicly available due to privacy or ethical restrictions.
